# Identifying predictors of resilient coping in students during COVID-19 lockdown

**DOI:** 10.1192/j.eurpsy.2024.256

**Published:** 2024-08-27

**Authors:** C. Laranjeira, A. I. Querido, M. A. Dixe

**Affiliations:** ^1^School of Health Sciences; ^2^ciTechCare, Polytechnic University of Leiria, Leiria, Portugal

## Abstract

**Introduction:**

Although increasing resilient coping throughout life is beneficial, it is particularly important in young people. To prevent the development of mental health problems, it is crucial to understand the factors associated with resilience. However, among university students, the characteristics considered conducive to resiliency have not been sufficiently studied, particularly during pandemic times.

**Objectives:**

The present study examined factors associated with resilient coping in Portuguese higher education students during the COVID-19 pandemic.

**Methods:**

Data were collected from an opportunity large sample of participants during the academic year 2020/2021. Four self-report measures were utilized within the study: Herth Hope Index, Brief Resilient Coping Scale, Depression Anxiety and Stress Scale – 21 items, and Impact of Event Scale-Revised. Additionally, a demographic questionnaire was used to collect data including age, gender, have children, education level, and study area. Ethics clearance was obtained. In order to test the research question, a multiple regression was conducted (using SPSS 28), with resilient coping as the dependent variable and the other variables entered as potential predictor variables.

**Results:**

A total of 1522 students (75.1% women and 24.9% men) took part in this study. Most participants were single (91.2%), had no children (93%), and the ages ranged from 18 to 59, with a mean age of 22.88±6.93 years. In terms of study level, the majority of students (73.7%) are at the undergraduate level and are not working (76.6%). Among the participants, 35.7%, 36.2%, and 28.5% had symptoms of stress, anxiety, and depression above the normal range, respectively. High resilience scores were found in 215 participants (14.1%). The mean hope (HHI) was 35.53 (SD = 5.92). Our results also demonstrated that hope is the only predictor of resilient coping (p<0.001). A higher level of hope is expected to affect people’s psychological adjustment by influencing both their appraisal of, and their coping with, the stressors confronted by them.

**Conclusions:**

Establishing and improving protective factors should increase the likelihood of the individual successfully avoiding negative outcomes and increase their ability to function normally, thus promoting resilient outcomes. We were able to draw practical implications for developing resilience-promoting methods in a university context. These results can be used to help students build resilience by preparing for future problems.

**Disclosure of Interest:**

None Declared

